# On the species status of the root-knot nematode *Meloidogyne ulmi* Palmisano & Ambrogioni, 2000 (Nematoda, Meloidogynidae)

**DOI:** 10.3897/zookeys.362.6352

**Published:** 2013-12-13

**Authors:** Mohammed Ahmed, Bart TLH van de Vossenberg, Chris Cornelisse, Gerrit Karssen

**Affiliations:** 1 National Plant Protection Organization, Wageningen Nematode Collection, P.O. Box 9102, 6700 HC Wageningen, The Netherlands; 2 Ghent University, Department of Biology, Ledeganckstraat 35, 9000 Ghent, Belgium

**Keywords:** Morphological, morphometrics, esterase, malate dehydrogenase, Japan, Italy, Mierenbos, *Malus prunifolia*, *Ulmus chenmoui*, SSU rDNA, LSU rDNA

## Abstract

The root-knot nematode *Meloidogyne ulmi* is synonymised with *Meloidogyne mali* based on morphological and morphometric similarities, common hosts, as well as biochemical similarities at both protein and DNA levels. *M. mali* was first described in Japan on *Malus prunifolia* Borkh.; and *M. ulmi* in Italy on *Ulmus chenmoui* W.C. Cheng. Morphological and morphometric studies of their holo- and paratypes revealed important similarities in the major characters as well as some general variability in a few others. Host test also showed that besides the two species being able to parasitize the type hosts of the other, they share some other common hosts. Our study of the esterase and malate dehydrogenase isozyme phenotypes of some *M. ulmi* populations gave a perfectly comparable result to that already known for *M. mali*. Finally, phylogenetic studies of their SSU and LSU rDNA sequence data revealed that the two are not distinguishable at DNA level. All these put together, leave strong evidences to support the fact that *M. ulmi* is not a valid species, but a junior synonym of *M. mali*. Brief discussion on the biology and life cycle of *M. mali* is given. An overview of all known hosts and the possible distribution of *M. mali* in Europe are also presented.

## Introduction

The genus *Meloidogyne* comprises all root-knot nematodes. It contains over 100 described species (Karssen et al. 2013). Its members are without a doubt the most widely distributed of all plant-parasitic nematodes (Sasser 1977). This widespread distribution and their economic importance are primarily the reasons why the genus has been the subject of more research than any other plant-parasitic nematodes, including the cyst-forming nematodes (Sasser and Carter 1982). Despite the numerous studies about their biology and taxonomy, their identification to the species level still pose a huge challenge to many diagnosticians (Blok and Powers 2009) mostly because of their very small inter-specific morphological variation (Jepson 1987).

In 2000, Palmisano and Ambrogioni described from Italy the root-knot nematode, *Meloidogyne ulmi*, from *Ulmus chenmoui* W.C. Chengon which it was found to induce large galls. For many years, elm remained the only known host of *Meloidogyne ulmi*. According to the authors, the tree at the type locality was introduced from the Netherlands as part of a breeding programme focussed on resistance to the Dutch elm disease. The Netherlands, like many other countries in Europe and North America, as well as New Zealand, has for years been battling against the notorious Dutch elm disease. It was for this reason that the former Dutch phytopathological laboratory “Willie Commelin Scholten” (WCS), based in Baarn, was mandated with the research on Dutch elm disease. This breeding programme was later on moved to Wageningen at the former institute the Dorschkamp Research Institute for Forestry & Landscape Planning; and in this programme, elm trees from all over the world were tested. Trial field “Mierenbos”, a part of the Dorschkamp Research Institute for Forestry & Landscape Planning, was used for growing and improving resistant elm cultivars. It was from this trial field that resistant elm seedlings were sent to ten other European countries at the end of the breeding programme, among others, Italy in 1992 (Heybroek 1993).

The first observation of galls on elm trees was already in 1960 at Baarn, and the associating nematode was diagnosed as *Meloidogyne arenaria* (Neal, 1889) Chitwood, 1949 by the National Plant Protection Organization (Oostenbrink 1961). Interestingly, about that same period, a *Meloidogyne* species found parasitizing apple trees in Japan was also inadvertently misidentified as *Meloidogyne arenaria*, because this nematode species, like the one on elm bore some resemblance to *Meloidogyne arenaria* perineal patterns (Itoh et al. 1969). This Japanese species would later be described and named *Meloidogyne mali* by Itoh et al. (1969). In his comprehensive study on the host range of *Meloidogyne mali*, Toida (1979) associated this species with several other plant species belonging to different families, particularly the Rosaceae. Following its description, several studies have also been conducted on its taxonomy, ecology, damage and control (Inagaki 1978), SEM studies of male and second-stage juvenile head morphology, and morphological variability of its different populations (Okamoto et al. 1983). At the trial field “Mierenbos”, the first report of galling symptoms on *Ulmus* trees was in 1979 (Brinkman 1980). Presently, all the *Ulmus* trees there are infected with *Meloidogyne* and are showing severe symptoms of root galling (Karssen et al. 2008 and Karssen 2009).

In 2006, root samples of the dying type host apple containing *Meloidogyne mali* from the type locality in Japan sent by Dr. Takayuki Mizukubo were received at the Dutch National Plant Protection organization. About the same time, galled root samples of the type host of *Meloidogyne ulmi* were obtained from Italy. They were propagated and maintained on the *Ulmus* × *hollandica* variety “Wredei”. Juveniles isolated from the Japanese apple root samples were used for sequencing and the resulting SSU rRNA sequence was discovered to be almost identical to that of *Meloidogyne ulmi* (Holterman et al. 2009). Additionally, isozyme phenotypes of *Meloidogyne ulmi* population from the trial field in Wageningen were also compared to that of *Meloidogyne mali* from Japan (Karssen unpublished; Sakai and Mizukubo 2009). Those also revealed similar patterns of esterase and malate dehydrogenase to that obtained for *Meloidogyne mali* (Sakai and Mizukubo 2009). With these observed similarities, a closer look needed to be taken into these two species. Based on the evidences available to us now, we hypothesize that *Meloidogyne ulmi* probably entered Europe as *Meloidogyne mali* with elm rootstocks imported from Japan. Supporting this is the report that *Meloidogyne mali*, in addition to its numerous hosts, can also infect *Ulmus davidiana* var. *japonica* (Toida 1979).

The original description of *Meloidogyne ulmi* differentiates it from *Meloidogyne mali* on the basis of characters that generally show high intraspecific variations. With the original description being the only paper written about *Meloidogyne ulmi*, all the known features so far are ones from the original description. On *Meloidogyne mali*, however, there have been quite a lot of research on the hosts, life cycle, ecology, detailed morphology, as well as their variations within species (Toida 1979; Inagaki 1978; Okamoto et al. 1983).

The objectives of this current research, therefore, are:

i) to evaluate the morphological similarities between *Meloidogyne mali* and *Meloidogyne ulmi*.

ii) to search for other host plants, than *Ulmus* sp. present at the trail field “Mierenbos”.

iii) to test *Meloidogyne ulmi* on selected host plants on which *Meloidogyne mali* is already known to reproduce.

iv) analyze their biochemical similarities, at the protein and DNA levels.

## Materials and methods

### Morphology and morphometrics

Paratype slides of *Meloidogyne ulmi* used for morphological and morphometric studies were obtained from Dr. Z.A. Handoo of the USDA Nematode Collection. In addition to these, we obtained *Meloidogyne mali* specimens on slides taken to the USDA by Dr. Ichinohe during his visit in 1958 as well as additional specimens of males, second-stage juveniles and females stored in formalin that were only recently isolated from root samples sent to USDA by Ichinohe during that same visit. Also, by courtesy of Dr. Hiromichi Sakai and Shigeyuki Sekimoto, paratypes that were deposited at the National Agriculture and Food Research Organization, Agricultural Research Center (Kannondai, Tsukuba, Ibaraki, Japan), the then Central Agricultural Experiment Station were also obtained. All slides were observed using compound light microscope (DM 2500, LEICA) equipped with differential interference contrast (DIC), and camera (DC 300F, LEICA) for taking images. Comparisons of morphological and morphometric characters were based on the most differential characters, previously used by Karssen (2002).

### Host test

This is a combination of sampling undertaken in 2011 and 2012 at the former trial field “Mierenbos” on several plant species and a subsequent greenhouse experiment involving some important plant species already associated with *Meloidogyne mali* in previous studies (Itoh et al. 1969; Toida 1979). Host herein is defined as a plant on which the nematode can reproduce, after a successful penetration.

### Isozyme analysis

Esterase and malate dehydrogenase isozymes were analysed for *Meloidogyne ulmi* sampled at “Mierenbos”, following the method described by Karssen et al. (1995). In summary, young females were isolated from roots into an isotonic (0.9%) salt solution. This was followed by a desalting step which involved transfer of the females from the NaCl solution to a reagent-grade water on ice for few minutes. Females were then singly transferred into sample wells containing 0.6 µl extraction buffer. With the aid of a small glass rod, the females in the wells were crushed; and the macerated females were then loaded into sample applicators (0.3 µl per well). All twelve wells, with the exception of 6 and 7, were loaded with our test samples of *Meloidogyne ulmi*. *Meloidogyne javanica* was used as reference in wells 6 and 7.

Electrophoresis was run using the PhastSystem (Pharmacia Ltd, Uppsala, Sweden) and the gels were subsequently stained in a Petri dish and placed in an incubator at 37 °C. Staining for non-specific esterase (EST; EC 3.1.1.1) was allowed to stand for 60 minutes whiles that for malate dehydrogenase (MDH; EC 1.1.1.37) stayed for 5 minutes.

Following staining, the gels were rinsed with distilled water and fixed for 5 minutes in a 10% acetic acid / 10 % glycerol/ 80 % distilled water solution. Pictures of the gels were taken by placing them on a glass surface illuminated from below.

### Molecular analysis

Already published sequences of both *Meloidogyne mali* and *Meloidogyne ulmi* (Holterman et al. 2009) together with our own sequence of the latter were included in this analysis.

### DNA extraction

Nucleic acids were isolated from single male or second-stage juveniles of *Meloidogyne ulmi* populations taken from “Mierenbos” and type populations kept in culture at the Dutch National Plant Protection Organization on an elm tree (*Ulmus* × *hollandica* Mill “Wredei”). Genomic DNAs were isolated from these samples using High Pure PCR Template Preparation Kit (www.roche-applied-science.com, Cat. No. 11796828001, Version 16.0) protocol for isolation of nucleic acids from Mammalian Tissue with slight modification in the first step to suit nematode DNA isolation (150 µl tissue lysis buffer added to 50 µl sterile water containing nematodes, minimum protease incubation time of 16 hours and elution volume of 50 µl).

### PCR and sequencing

Amplification of 1000 base pairs (bp) of the large ribosomal subunit (LSU) (28S) was performed using primer set 28–81for (forward) 5’-TTAAGCATATCATTTAGCGGAGGAA-3’ and 28–1006rev (reverse) 5’-GTTCGATTAGTCTTTCGCCCCT-3’ described by Holterman et al. (2008).

To amplify the nearly full length sequence of the small ribosomal subunit (SSU) (18S), two partially overlapping fragments were generated using three universal primers and one nematode-specific primer (1912R) described by Holterman et al. (2006). The latter’s inclusion was to avoid amplification of non-target eukaryotic SSU rDNA, for example from fungal spores attached to the nematode cuticle. The primers 988F (forward) (5’-CTCAAAGATTAAGCCATGC-3’) and 1912R (reverse) (5’-TTTACGGTCAGAACTAGGG-3’) were used to amplify the first fragment. The second fragment was amplified with primers 1813F (foward) (5’-CTGCGTGAGAGGTGAAAT-3’) and 2646R (reverse) (5’-GCTACCTTGTTACGACTTTT-3). Each PCR reaction mixture contained Molecular Grade Water (MGW)–DNase RNase free water (Sigma-Aldrich, Saint Louis, USA), 1x PCR buffer (incl. 2.0 mM MgCl_2_, Roche), dNTPs (0.2 mM each), 0.24 µM of each primer, FastStart Taq DNA polymerase (1 U/µl, Roche) and 1 µl of the template DNA. The final reaction volume was 25 µl. PCR was performed in C1000 touch thermal cycler (Bio-Rad) with the following amplification condition: 15 min at 95°C; 5 cycles of 30 sec at 94°C, 30 sec at 45°C and 30 sec at 72°C; 35 cycles of 30 sec at 94°C, 30 sec at 54°C and 30 sec at 72°C; final extension for 5 min at 72°C. To test for amplification and the quality of PCR products, 5 μl of the PCR products mixed with 1 µl 6x Bromophenol Blue Loading solution (Promega, Madison, USA) were subjected to electrophoresis and SYBR safe (Invitrogen, Carlsbad, USA) staining on a 1.5 % agarose gel by standard methods (Sambrook et al. 1989) along with a 1kb-plus DNA ladder (Invitrogen, Carlsbad, USA) to size fragments. PCR products were imaged under UV light using a GeneGenious gel imaging system (Syngene, Cambridge, United Kingdom).

PCR products were purified after amplification using QIAquick PCR Purification Kit (Qiagen), and the genomic DNA concentration measured using a ND1000 spectrophotometer (NanoDrop). This was followed by a cycle sequencing reaction in a final volume of 20 µl (molecular grade water (Sigma-Aldrich, Saint Louis, USA), BigDye Terminator v1.1, 1x sequencing buffer, purified PCR product and 0.5 µM template-specific forward or reverse primers). Cycling reactions were carried out separately for each of forward and reverse primers. The reaction programme was set for 1 min at 96°C, 25x (10 sec 96°C, 5 sec 50°C, 2.5 min 60°C), 1 min 20°C. The cycle sequence products were cleaned up using DyeEx 2.0 Spin Kit (Qiagen) and run on a multi-capillary 3500 Genetic Analyzer DNA sequencer (Applied Biosystems, Carlsbad, USA).

### Sequence alignment and phylogenetic analyses

Trace files of D2–D3 expansion segments of 28S and 18S-rRNA genes were assembled into contigs and amplification primer sequences trimmed using Geneouis 6.1.6 (Biomatters, New Zealand). Additional trimming was performed when needed, to obtain high quality consensus sequence data. Conflicts in the consensus sequence were assessed visually and corrected where possible. The sequences were aligned with selected sequences of other species from GeneBank using MAFFT alignment (Katoh et al. 2002) within the programme Geneious 6.1.6 (Biomatters New Zealand) for both 28S and 18S-rRNA. Alignments were improved manually. Analysis of phylogeny of the sequence data set was performed with Bayesian inference (BI) using MrBayes 3.2.1 (Huelsenback and Ronquist 2001). The optimal model for nucleotide substitution was obtained using JModelTest ver. 2.1.3 (Darriba et al. 2012) with AIC, AICc, BIC and DT defaulted in JModelTest. For SSU sequence, analysis of Bayesian inference was performed with a random starting tree and four Markov chains for 1 × 10^6^ under the model TYMef + I. Trees were sampled at interval of 100 generations. Two independent runs were performed for each analysis. The first 100,000 generations were discarded as burn-ins, and the remaining trees combined to generate 50% majority rule consensus tree which represent posterior probabilities. The same parameter settings were used for LSU phylogenetic analysis, but under the model TYM + G.

## Results

### Morphology

The following are the observations made on selected features considered to be the most differential for species discrimination among members of the genus *Meloidogyne* (Jepson 1987; Karssen 2002). Table 3 shows a comparison of our observations of the most important characters with the ones mentioned in the original descriptions.

**Table 1.** The various forms of type specimens of *Meloidogyne mali* and *Meloidogyne ulmi* studied and their sources.

Form	*Meloidogyne mali*		*Meloidogyne ulmi*	
	Sex/stage	Source	Sex/stage	Source
Holotype	1 female	Shigeyuki Sekimoto	–	–
Allotype*	1 male	Shigeyuki Sekimoto	–	–
Paratype	17 perineal patterns	Dr. Zafar A. Handoo	2 PP & 1 anterior part	Dr. Zafar A. Handoo
Paratype	3 males	Dr. Zafar A. Handoo	2 males	Dr. Zafar A. Handoo
Paratype	4 juveniles	Dr. Zafar A. Handoo	3 juveniles	Dr. Zafar A. Handoo
Paratype	1 male	Dr. Hiromichi Sakai	–	–
Paratype	1 juvenile	Dr. Hiromichi Sakai	–	–

*Slide marked as allotype, however at present not recognized by the ICZN, i.e. it is a paratype.

**Table 2.** Plant species included in the host plant test with *Meloidogyne ulmi* in the greenhouse.

Family	Plant species
Brassicaceae	*Brassica oleracea* L. var. Gemmifera (cabbage)
	*Brassica pekinensis* (Lour.) Rupr. (celery cabbage)
Rosaceae	*Malus pumila* “M9” (apple)
	*Rosa hybrida* L. (rose)
Fabaceae	*Trifolium repens* L. (white clover)
Solanaceae	*Solanum lycopersicum* L. (tomato)
Ulmaceae	*Ulmus glabra* Huds. (wych elm)

**Table 3.** Observations of the differential characteristics of female, male and second-stage juveniles of *Meloidogyne mali* and *Meloidogyne ulmi* types in comparison with their interpretation in the original description.

Species	*Meloidogyne mali*		*Meloidogyne ulmi*	
Character	Described	Observed	Described	Observed
**Female**				
Stylet knobs	Well developed knobs that tend to slope backward or forward in the ratio of 16 to 8	Rounded to pear-shaped knobs, set off and slightly anteriorly concave to backwardly sloping	Knobs rounded to transversely ovoid, slightly concave anteriorly	Rounded knobs that are slightly anteriorly concave and offset
Perineal pattern	Oval, made up of smooth striae, finely spaced, dorsal arch low and flat. Phasmids large, lateral field clearly marked with single or double incisures	Oval, dorsal arch low to slightly high, rounded to square shaped.<br/> Phasmids distinct. Lateral field marked by breaks in the striae or showing indistinct lateral lines	Oval, dorsal arch flattened to medium high, rounded or somewhat square, phasmids conspicuous, lateral field indistinct or marked by folds, sometimes by lateral lines on one or both sides	Oval, dorsal arch low to slightly high, rounded to square shaped.<br/> Phasmids distinct. Lateral field marked by breaks in the striae or showing indistinct lateral lines
**Male**				
Head shape	_	Head weakly offset, head cap low and slightly narrower than the postlabial region. Postlabial incisures absent	Head slightly set off, labial cap shallowly rounded, one-fifth to one-fourth as high as postlabial region	Head weakly offset, head cap low and slightly narrower than the postlabial region. Postlabial incisures absent
Stylet knobs	Knobs rounded	Backwardly sloping with rounded to pear shaped knobs	Knobs rounded to pear shaped more or less backwardly sloping	Backwardly sloping with rounded to pear shaped knobs
**Second-stage juvenile**				
Stylet knobs	Knobs backwardly sloped	Small rounded knobs, slightly backwardly sloping	Knobs rounded and set off from shaft	Small rounded knobs, backwardly sloping
Tail shape	Short	Conical with a broad to finely pointed tip	Conical, tapering to a finely rounded almost pointed terminus or broader and rounded at the tip	Conical and tapers to a broadly or finely pointed tip
Hyaline tail part	_	Constrictions present along hyaline part, length short or long. Anterior part clearly delimitated	Cuticular constrictions present along hyaline tail terminus, variable in length	Constrictions present along hyaline part, anterior part clearly delimitated

### Female

#### Perineal pattern

The general shape of the perineal pattern in both species studied ranged from low rounded to oval. The dorsal arch of *Meloidogyne mali* and *Meloidogyne ulmi* was mostly low rounded with very few instances where some specimens showed relatively high square patterns. Lateral field was marked by change in direction or breaks in striae resulting in what would appear as weak lateral lines. The double lateral lines mentioned in the description of *Meloidogyne mali* were not observed in the studied specimens. The interphasmidial distance in both species was about the same as their corresponding vulva slit lengths. As mentioned in the description, the phasmids were distinct but did not appear large when observed at the correct focus. However, attempting to observe them at the same (relatively deeper) focus as the vulva slit makes them look larger and even farther apart than they really are, due to the diagonally sloping phasmid canals.

#### Stylet

Same variations in stylet knobs shape as described in *Meloidogyne mali* were observed for *Meloidogyne mali* paratypes i.e. slightly backwardly sloping to anteriorly concave, with the former being the more frequent. Such variations, however, cannot be mentioned about the *Meloidogyne ulmi* paratype since there was only a single anterior part of the female on the slides we obtained. We therefore supplemented it with specimens taken from samples from the “Mierenbos”, where the type host originated from. This population showed similar variation as described for *Meloidogyne mali*, but not reported in Table 3. Our observation of the shape of the stylet itself was typical of the genus, i.e. straight shaft with a slightly dorsally curved cone.

#### Secretory-excretory pore

S-E pore position measured from the anterior end showed quite some variations. Nevertheless, all measurements taken for both species fell within the range described for *Meloidogyne ulmi*. This character in *Meloidogyne mali* description was measured on the basis of the number of annuli counted from the anterior end to the one bearing the S-E pore.

### Male

#### Head region

Under light microscope, both species have the same head outline. This was already illustrated in the descriptions of the two species (Itoh et al. 1969; Palmisano and Ambrogioni 2000). The head cap in both species is low. The presence of lip annuli mentioned in *Meloidogyne ulmi* was observed on some of the paratypes studied as well as in some of the additional specimens included later from “Mierenbos”. The post-labial cephalic region slightly set off from the remainder of the body. SEM observation of the *en face* view of the lip region was not part of this study. Nevertheless, this will be discussed further on in this work based on previous study conducted by Yaegashi and Okamoto (1981) as well as the original description of *Meloidogyne ulmi*.

#### Stylet

The stylet moderately slender. Conus with bluntly pointed tip. The shaft width the same along its entire length, although in some specimens it appeared to be broader close to the junction with the knobs. Individual knobs rounded to pear shaped. Knobs backwardly sloping in both species.

#### Lateral field

The lateral field marked by four incisures. In most of the specimens studied, the outer lines appeared areolated along most part of the body. No difference in the number of lateral incisures was observed along the body, except at the anterior part where it reduces to two and gradually fades out further anterior.

#### Hemizonid position relative to S-E pore

Although not considered to be of any diagnostic significance, this character remained fairly consistent in all specimens studied. The hemizonid always occurred anterior to the S-E pore, at slightly varying distances.

#### Second-stage juveniles

Examination of the second-stage juvenile characters was based on six *Meloidogyne mali* type specimens and two of *Meloidogyne ulmi*.

#### Head region

Head slightly set off from the rest of the body, with a low lip. Post-labial region lacking any annule.

#### Stylet

Stylet somewhat slender, with conus terminating in a fine tip, in both species. Stylet knobs small and rounded; slightly backwardly sloping.

#### Hemizonid position relative to S-E pore

Contrary to the condition in males, hemizonid always located behind the S-E pore in second-stage juveniles. However, the exact position is variable.

#### Tail

Tail mostly straight, ranging from short to medium; with a fine to bluntly rounded tip. Hyaline tail terminus with varying length, anterior part distinctly delimitated.

### Morphometrics

#### Females

Almost all our average measurements were within the range of those in the original descriptions (Tables 4–6). In the case of *Meloidogyne ulmi*, measured values of the female anterior part are based only on a single paratype specimen. Useful differential characters like the stylet length, stylet knob widths and stylet knob heights showed great similarities. From the perineal patterns, measurements of all the known important features also gave comparable values with those in the descriptions. Interphasmidial distance and the vulva slit were in most cases similar, rarely significantly different. In *Meloidogyne mali*, these two measurements were almost identical. There was however, a slight difference in these two measurements from *Meloidogyne ulmi* types (Table 4), probably because only two perineal patterns were studied.

**Table 4.** Morphometrics of *Meloidogyne mali* and *Meloidogyne ulmi* females in comparison with the original descriptions. All measurements are in µm and in the form: mean ± sd. (range).

Species	*Meloidogyne mali*		*Meloidogyne ulmi*	
Character	Described	Observed	Described	Observed
N	25	17	30	2*
Body length	847<br/> (684–1044)	762 ± 115<br/> (608–890)	771 ± 140<br/> (568–1043)	_
Body width	660<br/> (540–864)	570 ± 122<br/> (372–700)	618 ± 152<br/> (357–1007)	_
Neck length	166 ± 43.7<br/> (90–252)	165 ± 62<br/> (60–265)	165 ± 67<br/> (58–382)	205
Neck diameter	_	100 ± 34.3<br/> (48–160)	_	152
Stylet length	15<br/> (13–17)	11.9 ± 1.8<br/> (7.7–15.4)	14.2 ± 1.0<br/> (12.0–15.7)	13.4
Stylet knob height	_	1.6 ± 0.3<br/> (1–2.2)	1.8 ± 0.6<br/> (1.1–3.9)	1.6
Stylet knob width	_	3.2 ± 0.4<br/> (2.6–3.8)	3.5 ± 0.7<br/> (2.6–5.2)	3.2
DGO	5.5<br/> (4–7)	4.3 ± 1.5<br/> (2.2–6.7)	4.6 ± 0.8<br/> (3.3–6.5)	3.9
S-E pore	_	32.8 ± 5.5<br/> (25–43.5)	32.3 ± 7.8<br/> (15.7–45.1)	36.5
Metacorpus	110<br/> (90–147)	103 ± 7.9<br/> (90–117)	_	92.8
Metacorpus length	39<br/> (32–44)	40.4 ± 4.5<br/> (32–50)	42.6 ± 6.5<br/> (32.7–58.8)	39.7
Metacorpus diameter	49<br/> (40–73)	39.7 ± 6.5<br/> (29–47)	40.9 ± 7.0<br/> (31.3–59.0)	36.5
Metacorpus valve length	12<br/> (11–13)	13.0 ± 1.1<br/> (11.5–15.4)	12.4 ± 1.0<br/> (11.1–14.4)	17.9
Metacorpus valve width	10<br/> (9–11)	9.5 ± 1.3<br/> (7–11.2)	9.7 ± 1.4<br/> (7.2–12.4)	10.2
Vulva – anus distance	17 ± 1.8<br/> (14–22)	19.1 ± 2.6<br/> (12.8–22.4)	19.0 ± 1.9<br/> (15.0–22.2)	19.2 ± 0.8<br/> (18.8–19.8)
Interphasmidial distance	22 ± 3.5<br/> (17–29)	24.8 ± 4.7<br/> (17.6–35.2)	19.2 ± 3.8<br/> (13.7–28.9)	22.4 ± 4.5<br/> (19.2–25.6)
Level of phasmids to vulva	25 ± 2.4<br/> (19–31)	27.4 ± 2.8<br/> (24–33.9)	25.1 ± 4.2<br/> (15.7–39.2)	27.6 ± 0.9<br/> (26.9–28.2)
Level of phasmids to anus	_	8.1 ± 2.5<br/> (5.1–12.8)	6.9 ± 2.2<br/> (2.6–15.7)	8.7 ± 1.3<br/> (7.7–9.6)
Vulva slit length	18 ± 2.5<br/> (12–24)	24.4 ± 3.3<br/> (16–28.2)	22.0 ± 2.9<br/> (17–28.7)	24.5 ± 1.1<br/> (23.7–25.3)

*Two perineal patterns and a single anterior part.

**Table 5.** Morphometrics of *Meloidogyne mali* and *Meloidogyne ulmi* males in comparison with the original descriptions. All measurements are in µm and in the form: mean ± sd. (range).

Species	*Meloidogyne mali*		*Meloidogyne ulmi*	
Character	Described	Observed	Described	Observed
N	25	3	30	2
Body length	1447<br/> (1270–1630)	1428 ± 41.0<br/> (1380–1452)	1462 ± 190<br/> (1053–1776)	1455 ± 64<br/> (1410–15.0)
Body width	38<br/> (30–47)	34.8 ± 9.6<br/> (28.0–41.8)	36.9 ± 4.3<br/> (26.6–48.4)	40.5 ± 0.7<br/> (40.0–41.0)
Body width at stylet knobs	_<br/>	15.7 ± 0.4<br/> (15.4–16.0)	16.7 ± 1.3<br/> (14.5–19.4)	17.6 ± 1.1<br/> (16.8–18.4)
Body width at S.E pore	_<br/>	24.5 ± 6.1<br/> (20.2–28.8)	27.6 ± 2.6<br/> (23.0–31.5	28.4 ± 0.6<br/> (28.0–28.8)
Stylet length	20<br/> 18–22	19.9 ± 1.8<br/> (18.6–21.1)	19.4 ± 1.2<br/> (17.5–22.9)	19.9 ± 0.9<br/> (19.2–20.5)
Stylet knob height	_<br/>	_	2.4 ± 0.1<br/> (2.0–3.0)	_
Stylet knob width	_<br/>	_<br/>	3.9 ± 0.4<br/> (3.0–4.8)	_
DGO	8<br/> (6–13)	9<br/>	6.3 ± 0.8<br/> (4.8–8.5)	7.4 ± 0.5<br/> (7.0–7.7)
S–E pore*	_<br/>	135.2<br/>	147 ± 18.8<br/> (97–187)	139 ± 7.1<br/> 134–144
Metacorpus**	_<br/>	98 ± 21.5<br/> (83–114)	99 ± 9.8<br/> (76–119)	83 ± 12.7<br/> (134–144)
Spicule	32<br/> (28–35)	28.1 ± 10.3<br/> (20.8–35.3)	33.8 ± 1.9<br/> (30.0–37.5)	29.8
Gubernaculum	8.5<br/> (7–10)	10.1<br/>	9.0 ± 0.9<br/> (7.3–9.8)	8.4 ± 0.4<br/> (8.1–8.7)
Testis length	788<br/> (540–970)	803 ± 125.9<br/> (714–892)	716 ± 167<br/> (324–977)	752 ± 79<br/> (696–808)
T	55<br/> (34–65)	55.3 ± 8.6<br/> (49.2–61.4)	48.7 ± 9.7<br/> (27.9–71)	52 ± 3.2<br/> (49–54)

* Distance from anterior end to S-E pore.

** Distance from anterior end to valve plate of median bulb.

**Table 6.** Morphometrics of *Meloidogyne mali* and *Meloidogyne ulmi* second-stage juveniles in comparison with the original descriptions. All measurements are in µm and in the form: mean ± sd. (range).

Species	*Meloidogyne mali*		*Meloidogyne ulmi*	
Character	Described	Observed	Described	Observed
N	25	5	30	3
Body length	418<br/> (390–450)	420 ± 21.7<br/> (390–446)	413 ± 20.6<br/> (373–460)	384 ± 9.5<br/> (374–394)
Body width	14.5<br/> (14–16)	14.0 ± 1.1<br/> (12.2–15.2)	14.2 ± 1.8<br/> (12.1–18.2)	12.7 ± 3.8<br/> (8.6–16.9)
Body diameter at anus	8.5<br/> (7–9)	9.4 ± 1.8<br/> (8.3–11.5)	8.4 ± 1.0<br/> (7.3–10.9)	6.5 ± 0.7<br/> (6.0–7.0)
Stylet length	14<br/> (12–15)	12.1 ± 1.5<br/> (10.9–13.8)	10.0 ± 0.8<br/> (8.5–11.1)	11.1 ± 0.6<br/> 10.6–11.5
Tail length	31<br/> (30–34)	30.2 ± 4.3<br/> (24.3–33.9)	31.3 ± 3.1<br/> (24.2–37.5)	24.2 ± 0.8<br/> (23.4–25.0)
Tail terminus length	_	7.0 ± 2.1<br/> (5.1–9.8)	8.2 ± 1.8<br/> (4.8–12.7)	5.7 ± 1.1<br/> (4.5–6.7)
Anus–primordium	_	139 ± 11.4<br/> (125–152)	_	126 ± 21.8<br/> (111–151)
a	28.5<br/> (27–31)	30.2 ± 3.2<br/> (27.1–34.8)	29.5 ± 3.4<br/> (22.3–35.5)	32.5 ± 10.9<br/> (23.4–44.7)
c	13.3<br/> (12–15)	14.4 ± 2.3<br/> (12.5–17.4)	13.3 ± 1.2<br/> (11.5–16.6)	16.3 ± 0.7<br/> (15.8–16.8)
c’	3.7<br/> (3–5)	3.3 ± 0.7<br/> (2.5–3.9)	3.7 ± 0.5<br/> (2.5–4.7)	3.7 ± 0.5<br/> (3.3–4.1)

#### Males

Three male paratypes of *Meloidogyne mali* and two of *Meloidogyne ulmi* were measured. Some of the studied characters were only visible enough for measurement on single specimens, and therefore for such characters absolute values were taken rather than their averages. The stylet knobs widths and heights were examples of characters for which measurements were not taken on either species (Table 5) due to the fact that they appeared slightly degenerated on all slides, and so may give false measurements. Nevertheless there were still some outstanding similarities in the stylet length, spicule length and DGO between the observed and the described values.

#### Second-stage juveniles

Similar to the observations made in the females and the males, the second-stage juvenile morphometrics was very comparable in many features between the two species studied. There was, however an unaccountable difference between stylet length as described for *Meloidogyne mali* (14µm (12–15µm)) and that which was measured (12.1 ± 1.5µm (10.9–13.8µm)). Values of body width at anus level between the two descriptions were very similar. Somemeasurements taken from *Meloidogyne ulmi*, likewise were quite similar to those in the original descriptions, particularly, the Demanian ratios a and c’, while others such as stylet lengths showed slight differences (Table 6).

#### Host test

The ability of *Meloidogyne ulmi* to reproduce on various plant species was examined under greenhouse conditions. Host statuses of the various plants used in the greenhouse test are presented in Table 7. *Meloidogyne ulmi* population from “Mierenbos” used as inoculum was able to induce galls and reproduce on both *Ulmus glabra* and *Ulmus hollandica* ‘belgica’. The apple ‘M9’ also had galls which contained egg-laying females. Although galls were induced by *Meloidogyne ulmi* on *Brassica oleracea* var. *gemmifera*, most of these galls contained small non-gravid females whose development seemed to have ceased at some point. Therefore, it is herein not considered as a host. There were no galls on *Rosa hybrida* and the other cabbage species, *Brassica pekinensis*.

**Table 7.** Plants species identified as host of *Meloidogyne ulmi* from the green house experiments and field survey at “Mierenbos”.

Family	Plant species
**Greenhouse test**	
Ulmaceae	*Ulmus glabra* Huds.
	*Ulmus hollandica* ‘belgica’
Rosaceae	*Malus pumila* ‘M9’
Solanaceae	*Solanum lycopersicum* L.
**Field hosts**	
Sapindaceae	*Acer pseudoplatanus* L.
Balsaminaceae	*Impatiens parviflora* DC.
Asteraceae	*Taraxacum officinale* F.H. Wigg.
Dyopteridaceae	*Dryopteris filix-mas* (L.) Schott
	*Dryopteris carthusiana* (Vill.) H.P. Fuchs
Fagaceae	*Fagus sylvatica* L.
	*Quercus robur* L.
Geraniaceae	*Geranium robertianum* L.
Rosaceae	*Geum coccineum* Lindl.
	*Rubus idaeus* L.
	*Sorbus aucuparia* L.
Taxaceae	*Taxus baccata* L.
Ulmaceae	*Ulmus davidiana* var. *japonica* Rehder
Urticaceae	*Urtica dioica* L.

**Table 8.** A compilation of all known host plants of *Meloidogyne mali* to date.

Family	Plant species	Reference
Rosaceae	*Malus pumila* Mill.	Itoh et al. 1969
	*Malus prunifolia* Borkh.	Itoh et al. 1969
	*Malus sieboldii* Rehd.	Itoh et al. 1969
	*Malus pumila* “M9”	Current work
	*Prunus yedoensis* Matsum	Itoh et al. 1969
	*Rosa hybrida* Hort.	Itoh et al. 1969
	*Geum coccineum* Lindl.	Current work
	*Vitis vinifera* L.	Itoh et al. 1969
	*Rubus idaeus* L.	Current work
	*Sorbus aucuparia* L.	Current work
Moraceae	*Morus bombycis* Koidz.	Itoh et al. 1969
	*Ficus carica* L.	Toida 1979
	*Maclura tricuspidata* (Carriere) Bureau	Toida 1979
	*Broussonetia papyrifera* (L.) Vent	Toida 1979
	*Broussonetia kazinoki* Seibold.	Toida 1979
Fagaceae	*Castanea crenata* Seib. Et Zucc	Itoh et al. 1969
	*Fagus sylvatica* L.	Current work
	*Quercus robur* L.	Current work
Ulmaceae	*Ulmus davidiana* var. *japonica*	Toida 1979
	*Ulmus chenmoui* W.C. Cheng	Palmisano and Ambrogioni 2000
	*Ulmus glabra* Hud.	Palmisano and Ambrogioni 2000
	*Ulmus* × *hollandica* “belgica”	Current work
Sapindaceae	*Acer palmatum* Thunb.	Itoh et al. 1969
	*Acer pseudoplatanus* L.	Current work
	*Trifolium repens* L.	Itoh et al. 1969
Taxaceae	*Taxus baccata* L.	Current work
Fabaceae	*Impatiens parviflora* DC.	Current work
Solanaceae	*Solanum lycopersicum* L.	Toida 1979
	*Solanum melongena* L.	Toida 1979
	*Capsicum annuum* L.	Toida 1979
Cucurbitaceae	*Cucumis sativus* L.	Toida 1979
	*Cucurbita* spp.	Toida 1979
	*Citrillus vulgaris* Schrad. Ex Eckl. & Zeyh.	Toida 1979
Cruciferae	*Brassica pekinensis* Rupy.	Toida 1979
	*Brassica oleracea* var. *capitata* L.	Toida 1979
	*Brassica napus* var. *oleifera* L.	Toida 1979
Compositae	*Arcutium lappa* L.	Toida 1979
	*Taraxacum officinale* F.H. Wigg.	Current work
Umbelliferae	*Daucus carota* var. *sativa* L.	Toida 1979
Leguminaceae	*Glycine max* (L.) Merr.	Toida 1979
Urticaceae	*Urtica dioica* L.	Current work
Dryopteridaceae	*Dryopteris filix-mas* (L.) Schott	Current work
	*Dryopteris carthusiana* (Vill.) H.P. Fuchs	Current work
Geraniaceae	*Geranium robertianum* L.	Current work

Additionally, samples collected during 2011 and 2012 revealed that *Meloidogyne ulmi* is able to parasitize one or more species of *Acer* (Aceraceae), *Impatiens* (Balsaminaceae), *Taraxacum* (Compositae), *Dryopteris* (Dryopteridaceae), *Fagus* (Fagaceae), *Quercus* (Fagaceae), *Geranium* (Geraniaceae), *Geum* (Rosaceae), *Rubus* (Rosaceae), *Sorbus* (Rosaceae), *Taxus* (Taxaceae), *Urtica* (Urticaceae), as shown in Table 7.

#### Isozyme analysis

Samples taken from the trial field “Mierenbos” all gave the same type of esterase isozyme pattern of weak single bands, corresponding to the VS1 type (Esbenshade and Triantaphyllou 1985). When analysed for MDH, some individuals gave single-banded patterns of the H1 type (Esbenshade and Triantaphyllou 1985), while others revealed a three-banded pattern, herein designated H3. Usually, the H1 type had two additional weaker bands at the same level as the upper two H3 bands. There was also an additional observation in the types of single bands some of the specimens produced (Fig. 3). These single bands were positioned at the same level as the upper H3 band, which herein are given the name H1a.

**Figure 1.**
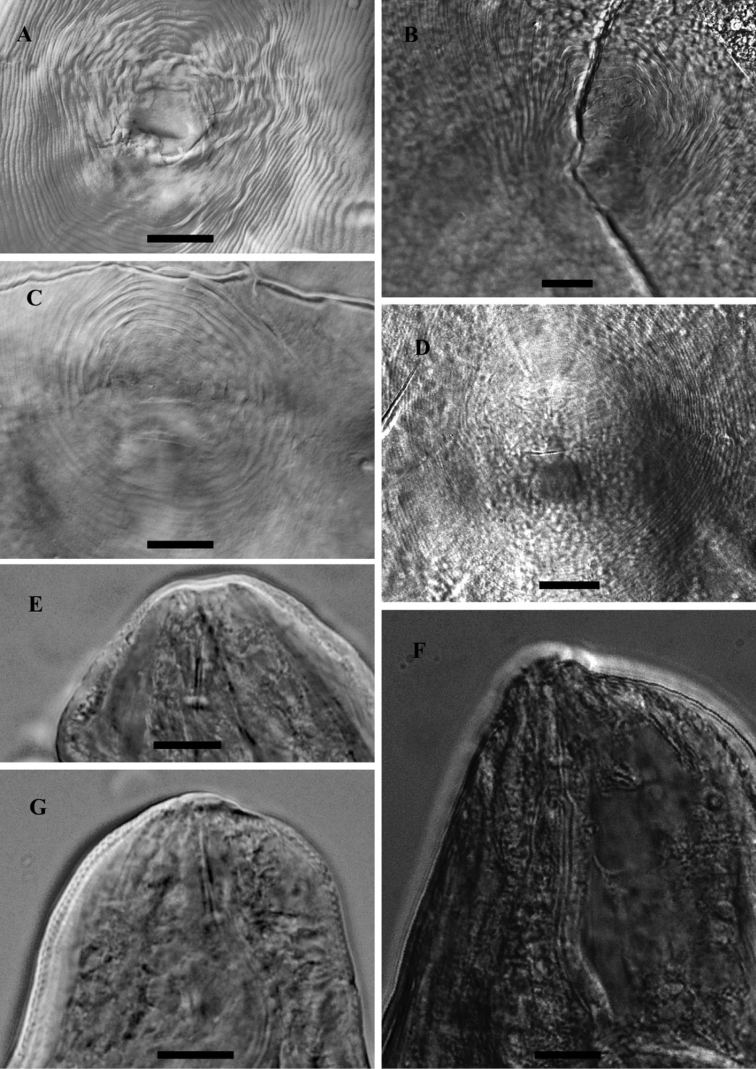
LM photograph of perineal patterns and anterior parts of female *Meloidogyne mali* (**A, C, E, G**) and *Meloidogyne ulmi* (**B, D, F**), bar = 10 µm.

**Figure 2.**
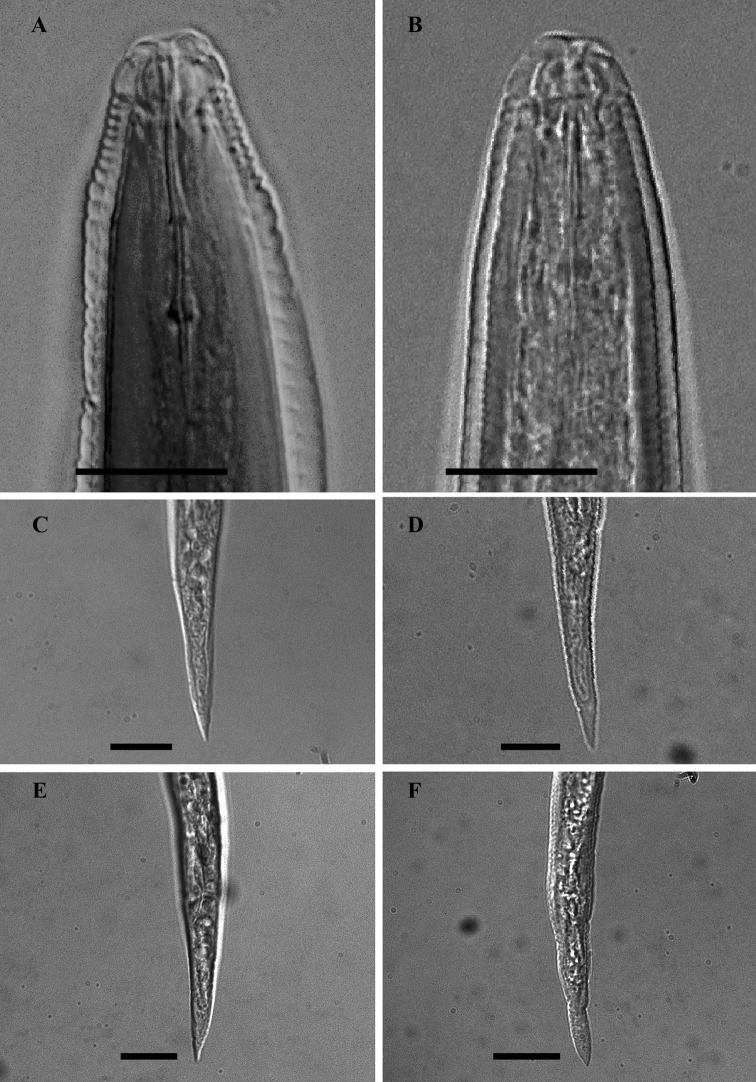
LM photographs of males anterior part and second- stage juvenile tails of *Meloidogyne mali* (**A, C, E**) and *Meloidogyne ulmi* (**B, D, F**), bar = 10 µm.

**Figure 3.**
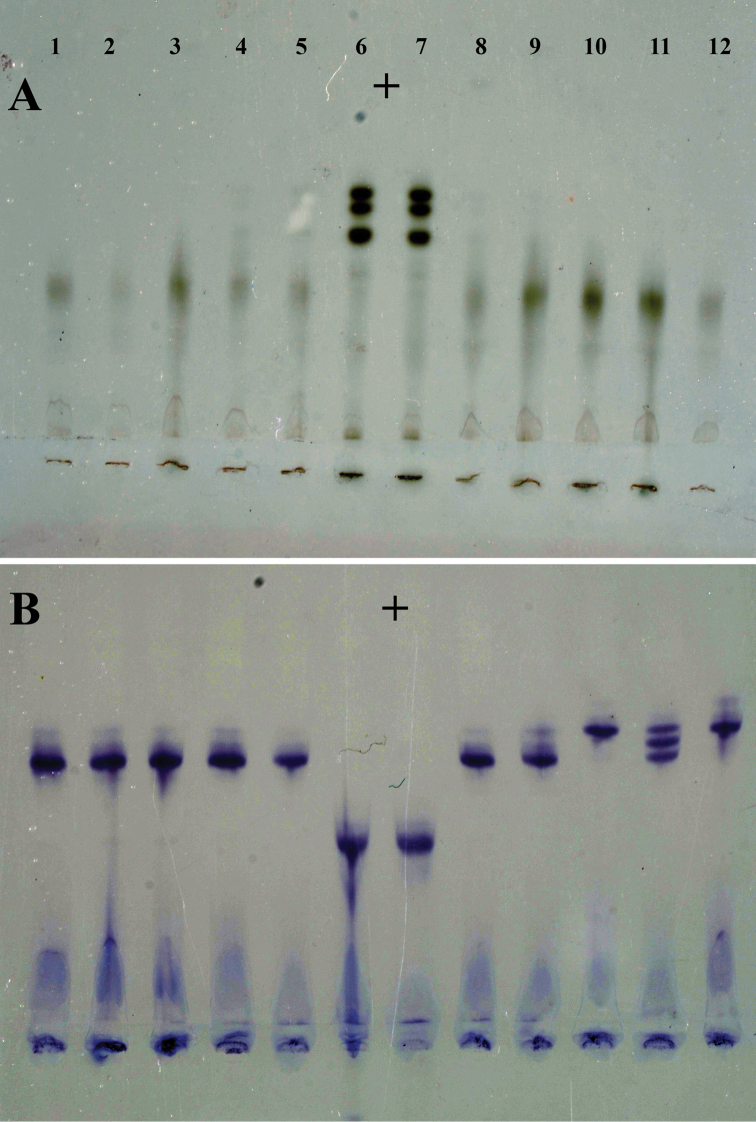
Isozyme phenotypes from ten individual females of *Meloidogyne ulmi* from “Mierenbos”. **A** Esterase **B** Malate dehydrogenase. *Meloidogyne ulmi* (1–5 and 8–12); *Meloidogyne javanica* (6 and 7) as reference marker.

### Phylogenetic relationship between *Meloidogyne mali* and *Meloidogyne ulmi*

The obtained SSU rDNA and LSU rDNA sequence lengths for *Meloidogyne ulmi* were 781bp (including gaps) and 698bp (including gaps) respectively. In addition to our four SSU rDNA sequences of *Meloidogyne ulmi* “Mierenbos” (KF895397, KF895398, KF895399 and KF895400), 69 accessions belonging to other species of *Meloidogyne* from GeneBank were included in the local alignment (781 aligned positions, including gaps). For LSU rDNA, we had only one sequence of *Meloidogyne ulmi* “Mierenbos” (KF895396) due to poor data, resulting in lack of consensus sequence. Therefore the multiple sequence alignment included this sequence and 69 GeneBank accessions from other species of *Meloidogyne*. *Pratylenchus vulnus* Allen & Jensen, 1951 was selected as outgroup for constructing gene trees using Bayesian inference from both SSU rDNA and LSU rDNA sequences. SSU rDNA-based phylogenetic analysis put all sequences of *Meloidogyne ulmi* obtained together with those of *Meloidogyne mali* and *Meloidogyne ulmi* from GeneBank in one strongly supported polytomous branch. Despite the relatively short sequence length of SSU rDNA, the tree was able to resolve relationship between certain species in a way comparable with that of Holterman et al. (2009). LSU rDNA-based Bayesian analysis revealed higher resolution within the group containing our sequence and sequences of *Meloidogyne mali* and *Meloidogyne ulmi* from GeneBank. Our sequence of *Meloidogyne ulmi* was positioned in a branch that contained three other sequences of *Meloidogyne mali*, forming a sister group to another branch composed also of two sequences of *Meloidogyne ulmi* and one of *Meloidogyne mali*. As would be expected, there was higher resolution in the overall topology of LSU rDNA-based tree than that of SSU rDNA. The sequence identity between the closest sequences of *Meloidogyne mali* and *Meloidogyne ulmi* was 98.8 % with one gap. Interestingly, the same percentage was obtained between two sequences of *Meloidogyne mali* from GeneBank (JX978226 and JX978227).

## Discussion

Type specimens representing holotypes and paratypes of the two *Meloidogyne* species were analysed in order to demonstrate the morphological similarities that existed between them. Most of the slides we received were in good conditions except for some few individuals that showed some signs of deterioration, either due to long period of storage or poor conditions at the time they were prepared. Nevertheless, the general states of the important characters were still maintained.

On the morphology, an important character like the occurrence of double lateral lines mentioned in the description and by Jepson (1987) was not observed in all specimens. In fact this area was marked by breaks in the striae on some of the specimens studied. Morphometric values of some characters for the two species fell within the range of values reported in the original description of *Meloidogyne mali* (Itoh et al. 1969), for example position of DGO in females, as well as in males, vulva-anus distance, level of phasmids to vulva, vulva slit length, male stylet length, testis length, spicule length gubernaculum length, J2 body length, and J2 c’ values (Tables 4–6). For some characters, however, morphometric values recorded agreed more with those reported for *Meloidogyne ulmi*. For example, the stylet length measured in females and J2s was significantly lower than the values given for *Meloidogyne mali*, but comparable with that of *Meloidogyne ulmi*. A possible explanation might be the fact that stylet in juveniles and sometimes in females appear less visible, causing the anterior end to be mistaken for the tip of the conus. This leads to misleadingly higher values for the stylet length.

Already in the original description of *Meloidogyne ulmi*, only a few differences could be found to separate it from *Meloidogyne mali*. And in some cases, the differences emanated from some apparent mistakes in the original description of *Meloidogyne mali*. An example is the use of male tail length of the two species to draw differences. Tail length in males of *Meloidogyne mali* was given as ranging between 28 and 44 µm, making it extremely longer than that of *Meloidogyne ulmi* 10.9 µm. However, it is important to mention that tail length values as long as the range of between 28 and 44 µm never exists among males of species of the genus *Meloidogyne*.

The DGO position in males with reference to the stylet knobs according to Jepson (1987) bears some broad interspecific variation, making it very useful for species discrimination. *Meloidogyne mali* is by far the species with the most farther DGO position (6–13 µm) within the genus *Meloidogyne*. The observation of similar values for both species studied here therefore separates the two from all other species that have relatively shorter DGO position. Additional *Meloidogyne ulmi* specimens studied also gave DGO position values averaging 8 µm (data not shown).

Eisenback and Hirschmann (1981) highlighted the significance of SEM studies of male head shapes in *Meloidogyne* taxonomy, outlining the role SEM has played in raising the value of males for use in comparison of species. Head and stylet shape morphologies of males and juveniles are the most useful supplemental taxonomic characters that SEM studies have given new insights into (Eisenback and Hirschmann 1979; 1981). It is not surprising that a number of variability in these characters were outlined to separate *Meloidogyne ulmi* from *Meloidogyne mali* by Palmisano and Ambriogioni (2000). It was mentioned (p. 288) that “under SEM lateral lips absent or vestigial (in *Meloidogyne mali* lateral lips apparent)”. Interestingly, this contradicted the comparison made by Toida and Yaegashi (1984) when they attempted to point out the differentiating characters between *Meloidogyne suginamiensis* and *Meloidogyne mali*. In their comparison, the *enface* view of the lip region of *Meloidogyne mali* was mentioned as having no or obscure lateral lips to separate it from *Meloidogyne suginamiensis* in which lateral lips were described as clear. One would not expect such contradicting accounts especially with the possibility that both works referred to the same publication (Okamoto et al. 1983). Referring to a separate work (Yeagashi and Okamoto 1981), the account given by Toida and Yaegashi (1984) seems to us more probable, the first reason being that they published the same work which is being referred to. Therefore they understand the details of their results more. And the second reason is that our observations of SEM images of (Yeagashi and Okamoto 1981) agree more with the account that lateral lips were vestigial and not apparent in *Meloidogyne mali*.

Both apple and elm trees supported *Meloidogyne ulmi* reproduction. This does not only provide an additional support for the synonymization of *Meloidogyne ulmi* with *Meloidogyne mali*, but represents the first and only test involving the former on an apple plant. In principle, however, the first actual report was the description of *Meloidogyne mali* on apple in Japan (Itoh et al. 1969). Contrary to the finding in the original description, the status of white clover as host to *Meloidogyne mali* could not be confirmed with *Meloidogyne ulmi*. Again, although representatives of the family Rosaceae form the larger part of the plants *Meloidogyne mali* parasitizes (Itoh et al. 1969; Toida 1979), rose (*Rosa hybrida*) could not support the reproduction of *Meloidogyne ulmi*. This contradicts earlier finding, Itoh et al. 1969, who identified rose as host. *Meloidogyne ulmi* was also able to induce galls on *Prunus yodoensis* grown in the field, confirming the earlier report of the latter’s status as a host for *Meloidogyne mali* by Toida (1979). Results of the sampling have also revealed new natural hosts for *Meloidogyne ulmi* like *Acer pseudoplatanus* L., *Fagus sylvatica* L., *Quercus robur* L., *Rubus idaeus* L., *Sorbus aucuparia* L., *Taxus baccata* L., *Dryopteris filix-mas* (L.) Schott, *Dryopteris carthusiana* (Vill.) Fuchs, *Geranium robertianum* L., *Urtica dioica* Rehder., *Impatiens parviflora* DC., *Taraxacum officinale* F.H. Wigg., and *Geum coccineum* Lindl. The most relevant evidence deduced from this host plant test is the ability of *Meloidogyne ulmi* to reproduce on apple.

It is interesting to mention that the observed variability of the MDH isozyme phenotypes among the different specimens was similar to the findings of Sakai and Mizukubo (2009) when they studied two populations of *Meloidogyne mali* from Hokkaida on apple and Saitama on cherry in Japan. The populations from Hokkaida gave phenotypes with single MDH bands whereas those from Saitama on cherry produced variable patterns with single and triple bands. PCR-RFLP of D2/D3 expansion segment of 28S rDNA and mtDNA intergenic region with *Alu* I was able to confirm that population from Saitama were all identical, despite their expression of variable MDH isozyme phenotypes. Similar observations of intraspecific phenotype variations were made by Dalmasso and Bergé (1978) among a certain *Meloidogyne arenaria* population where there were three MDH bands instead of two. Such type of variable isozyme patterns were also observed within one population of the sexually reproducing species *Meloidogyne microtyla* Mulvey, Townshend & Potter, 1975 (Karssen unpublished). This indicates that *Meloidogyne mali* could also be a sexually reproducing species or a meiotic parthenogenetic one, a claim which is further supported by the frequency at which males are encountered in galled root samples—at least one male per female in a gall. Meanwhile, the esterase phenotypes were rather stable across all studied specimens and were characterised by weak indistinct single bands.

**Figure 4.**
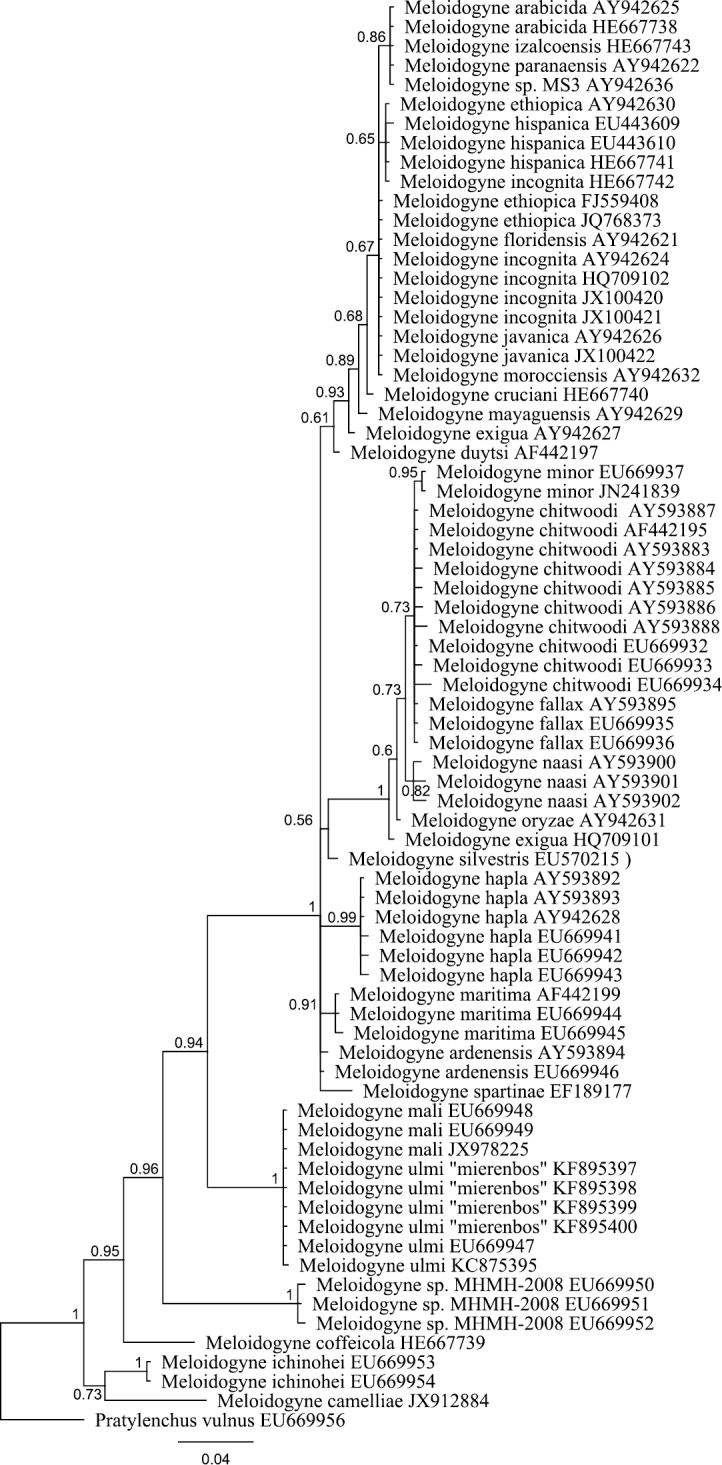
Bayesian tree inferred from part of 18S rRNA using TYMef + I model. Sequences were aligned with MAFFT alignment. Numbers near the nodes indicate posterior probabilities. NCBI accession numbers are listed with the species names.

Trimming the SSU and LSU datasets to high quality sequence data may have caused a loss in phylogenetic signal. For SSU rDNA, over half of the target sequence length was trimmed out because of the poor quality of the dataset obtained. Although not ideal for reconstruction of phylogeny, it was still sufficient to resolve the taxa on a species level. Moreover, it has to be emphasized that the purpose here is not to reconstruct any formal phylogeny of *Meloidogyne*, a subject which is well covered already in previous studies (Tandingan De Ley et al. 2002; Holterman et al. 2009), but only to demonstrate that *Meloidogyne mali* and *Meloidogyne ulmi* are highly similar at the molecular level and belong to the same clade. A recent phylogenetic analyses involving these two species has already pointed to the fact that the two can not be separated based on their SSU rDNA sequences (Rybarczyk-Mydłowska et al. in press). The SSU rDNA sequence once again gave resolution till the species level, confirming previous proposition that SSU rDNA sequence signatures can be deﬁned at species level for a wide range of parasitic and non-parasitic nematodes (Holterman et al. 2006). As was expected, LSU rDNA-based analysis gave even higher resolution and more clearly defined the relationship between *Meloidogyne mali* and *Meloidogyne ulmi*. On the SSU rDNA based tree, it is unquestionable that our sequence of *Meloidogyne ulmi* with all the other sequences of *Meloidogyne mali* and *Meloidogyne ulmi* are the same (Fig. 4). The clustering of our sequence of LSU rDNA for *Meloidogyne ulmi* with those for *Meloidogyne mali* may be an indication that the branching could only be due to intraspecific sequence variation.

## Conclusion

In conclusion, the evidence from morphological and morphometrical studies of holo– and paratype materials of *Meloidogyne mali* and *Meloidogyne ulmi* as well as host plant studies, isozyme analysis and DNA analysis all confirm the status of *Meloidogyne ulmi* as a junior synonym of *Meloidogyne mali*.

### Notes on the life cycle and biology of *Meloidogyne mali* on *Ulmus* spp.

The life cycle of *Meloidogyne mali* is in many respects typical of the genus. *Meloidogyne mali* requires 18–22 weeks to complete one full generation on apple and does so only once in a year (Inagaki 1978). The study also reported that adult males and females first were observed after twelve weeks and continued to increase till the twentieth, when egg masses began to appear. There was also some reports on the distribution of *Meloidogyne mali* in the field, both vertically and horizontally. However, nothing is known so far about its survival on apple or any other plant during frost conditions of winter. Regarding this, a very interesting observation was made during early spring of 2013 at the trial field “Mierenbos”. Egg-laying females were already found in most galls that were examined, a rare phenomenon known to occur only in *Meloidogyne ardenensis* (Stephan and Trudgill 1982). The only plausible explanation to why egg-laying females can be observed so early in the year is that, like reported for *Meloidogyne ardenensis*, the nematodes overwintered in the roots. Additional observations need to be made to find out exactly what stage in the development overwinters in the root.

### Host plants and distribution of *Meloidogyne mali* in Europe

*Meloidogyne mali* induces a similar type of galls as do *Meloidogyne arenaria* on tomatoes, a type of gall commonly referred to as bead-like galls (Fig. 6). Concerning the current distribution of the nematode in Europe, no study has yet been done to investigate this. However, it would be rational to speculate that *Meloidogyne mali* may be found in all the ten European countries to which rooted seedlings were sent after the breeding programme. These countries include Belgium, England, France, Ireland, Italy, Spain, Denmark, Germany, Slovakia and Romania (Heybroek 1993). Elsewhere in Asia, it has also been found in *Acer palmatum* trees from Japan that were intercepted in China (Gu unpublished). Sequence data from these were also included in the analysis.

**Figure 5.**
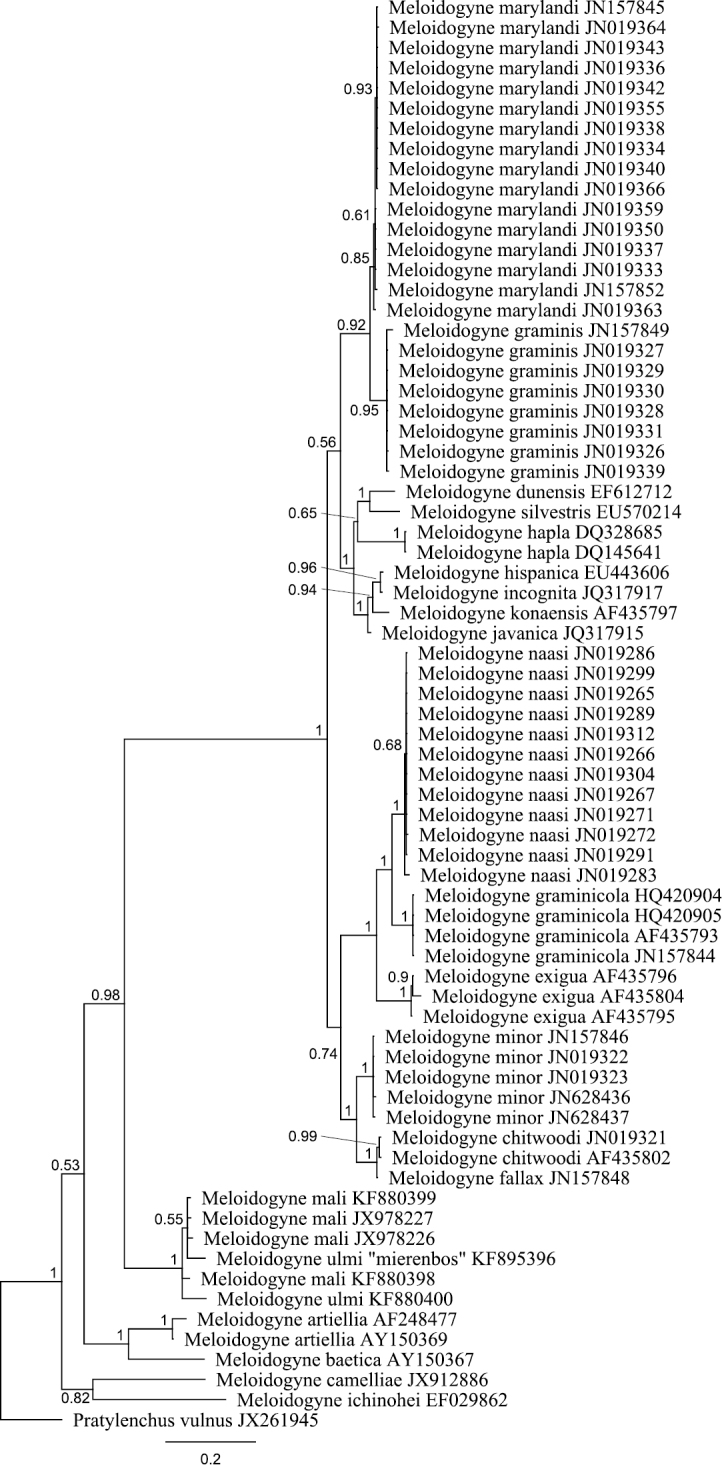
Bayesian tree inferred from part of D2 D3 of 28S rRNA using TYM + G model. Sequences were aligned with MAFFT alignment. Numbers near the nodes indicate posterior probabilities. NCBI accession numbers are listed with the species names.

**Figure 6.**
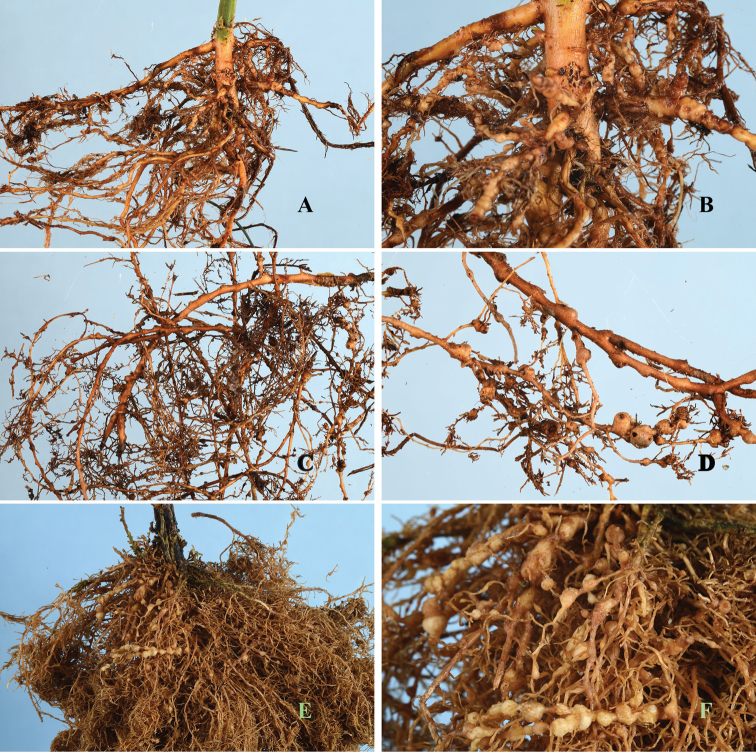
Root gall symptoms of *Meloidogyne mali* infection on (**A, B**) *Maluspumila* “M9” (**C, D**) *Ulmus davidiana* var. *japonica* and (**E, F**) *Solanum lycopersicum*.
